# Death of Dr. S. F. Starley

**Published:** 1888-01

**Authors:** 


### DEATH OF DR. S. F. STARLEY, OF TYLER.

In the death of this estimable gentleman and able physician, the whole State of Texas has suffered a severe loss, and the State Medical Association has lost one of its oldest, ablest and most zealous co-laborers. But three short years ago, Dr. Starley, then, though past 60 years, in the prime and vigor of manhood, presided with dignity over the deliberations of that body at Belton, and contributed much to the eclat which has caused that meeting to occupy a brilliant page in the medical history of Texas.

Dr. Starley was holder of certificate No. 25 in the Physicians' Mutual Benefit Association of Texas, he being one of its promoters and lasting friends, and assessments have been issued for the benefit of his family. A report of the results will be published in the March number of THE JOURNAL. It is a little singular, in this connection, that Dr. Park, of Tyler, who was the first member of the Order who died, held No. 26, and Dr. Starley, also of Tyler, the fifth death, was holder of No. 25,—or that two of the five deaths should have been among the profession at Tyler, and holders of consecutive numbers in the Order.

Our purpose of writing an obituary of our deceased friend has been so entirely anticipated by the profession of Tyler, who held Dr. Starley in high esteem, that we reproduce here the report of the meeting of the physicians on the occasion of his death.

